# Long-term Helicobacter pylori infection is associated with an increased risk of carotid plaque formation: a retrospective cohort study

**DOI:** 10.3389/fcvm.2024.1476435

**Published:** 2024-10-24

**Authors:** Yi Chen, Bingqian Ni, Chaoyu Yang, Jingjing Pan, Jinshun Zhang

**Affiliations:** ^1^ Department of Gastroenterology, Taizhou Hospital of Zhejiang Province Affiliated to Wenzhou Medical University, Taizhou, China; ^2^ Department of Otolaryngology, Taizhou Hospital of Zhejiang Province Affiliated to Wenzhou Medical University, Taizhou, China; ^3^ Zhejiang Provincial Centers for Disease Control and Prevention, Hangzhou, China; ^4^ Home Ward, Taizhou Hospital of Zhejiang Province Affiliated to Wenzhou Medical University, Taizhou, China; ^5^ Health Management Center, Taizhou Hospital of Zhejiang Province Affiliated to Wenzhou Medical University, Taizhou, China

**Keywords:** helicobacter pylori, carotid plaque, cohort study, blood fat, persistent infection

## Abstract

**Background:**

Cardiovascular disease significantly impacts human health. The development of carotid plaques elevates the risk of cardiovascular disease, while the influence of Helicobacter pylori (H. pylori) on carotid plaques remains a subject of debate. This study aimed to investigate the association between H. pylori infection and carotid plaque using a cohort study.

**Methods:**

The study included individuals who underwent multiple physical examinations at the Health Examination Center of Taizhou Hospital. The relationship between H. pylori and carotid plaque was explored using multifactorial logistic regression analysis. Participants were categorized into groups based on their H. pylori infection status at the initial and final examinations, comprising persistent infection, persistent negative, new infection, and eradication infection, to analyze variations in carotid plaque prevalence among these groups.

**Results:**

In both univariate and multifactorial regression analyses, H. pylori was identified as a risk factor for carotid plaque development. Moreover, when compared to the persistent negative group, both the new infection and persistent infection groups showed a notable increase in the risk of carotid plaque. Additionally, individuals in the persistent infection group exhibited higher blood pressure and blood glucose levels than those in the persistent negative group. Likewise, there was a discrepancy in the impact of insulin resistance on carotid plaque between the H. pylori positive and negative groups.

**Conclusion:**

H. pylori is a risk factor for carotid plaque, with a long-term infection associated with an increased risk of carotid plaque formation. In addition, H. pylori promoting carotid plaque formation may be related to blood pressure, blood glucose, and insulin resistance.

## 1 Introduction

Helicobacter pylori (H. pylori) infection is a global public health issue with a higher prevalence in developing countries (1). H. pylori attaches to the host gastric epithelium through various adhesins, secretes multiple virulence factors, modulates cell signaling, and induces an inflammatory response (2). This infection can cause localized inflammation of the gastric mucosa, resulting in digestive disorders, atrophic gastritis, gastric cancer, and related illnesses (3–5). Nowadays, more and more studies focus on studying the impact of H. pylori on extragastric diseases, including liver, respiratory system, diabetes, hematologic, and cardiovascular diseases (6–8).

Atherosclerosis, a chronic inflammatory condition, presents a significant risk to human health (9). It can lead to diffuse intimal thickening, arterial calcification, and the development of vulnerable plaques prone to rupture, ultimately culminating in the complete occlusion of the vessel wall (10). Carotid intima-media thickness (IMT) and carotid plaque are important references for the assessment of atherosclerosis (11). The composition and stability of carotid plaques are important danger factors for acute cardiovascular and cerebrovascular events (12). The process of atherosclerosis involves vascular inflammation, immune response, thrombosis and other mechanisms (13). H. pylori stimulates the release of cytotoxin-associated gene A (CagA), leading to the promotion of atherosclerosis and plaque formation via immune responses and inflammatory reactions (14, 15). Additionally, insulin resistance (IR) significantly influences atherosclerosis progression, and H. pylori infection may worsen this impact (16, 17). Triglyceride glucose (TyG) index is surrogate for IR and is increasingly being used in cardiovascular disease (18, 19).

While there is mounting concern regarding the connection between H. pylori and cardiovascular disease, the relationship with carotid plaque remains contentious (14, 20, 21). Most studies have employed cross-sectional methodologies to investigate the correlation between H. pylori and carotid plaque. In this research, we carried out a comprehensive cohort study to investigate the relevance of H. pylori in carotid plaque development within a Chinese medical examination population.

## 2 Materials and methods

### 2.1 Study population

This study included individuals who underwent medical check-ups at the Taizhou Hospital Medical Examination Center from 2017 to 2022. Participants with complete clinical information, such as age, gender, smoking, drinking, laboratory parameters, urea breath test results, blood pressure, and neck ultrasound data, were required for the study population. Laboratory parameters assessed comprised fasting blood glucose (FBG), glycated hemoglobin A1c (HbA1c), triglyceride (TG), low-density lipoprotein (LDL), total cholesterol (TC), high-density lipoprotein (HDL). Exclusion criteria identified patients with current pregnancy, a history of gastrointestinal surgery, thyroid disorders, malignancies, or insufficient clinical data. Each participant underwent multiple medical check-ups, spaced six months apart from the first to the last assessment. The study included a total of 5,994 individuals for follow-up analysis.

### 2.2 Carotid plaque measurement

All participants underwent ultrasound examinations of the bilateral common carotid arteries, carotid bifurcations, and internal carotid arteries using a 7–12 MHz scanning frequency B-mode ultrasound machine (Sonos 5,500; Agilent, Santa Clara, CA). Subjects adopted a low occipital supine posture, tilting their heads backward and leaning towards the unexamined side to adequately reveal the neck. A skilled sonographer used a ultrasound device to assess the IMT in the neck vessels. Plaques were identified as regions where the IMT exceeded 50% of the surrounding areas (22).

### 2.3 Clinical indicators collection

Following an overnight fasting period of 8 h, blood was drawn from participants to measure laboratory parameters including TC, HDL, LDL, TG, FBG, and HbA1c. Both laboratory tests and ultrasounds were carried out on the same day. Adequately trained nurses initially gathered the participants’ age, gender, smoking and drinking history, and personal medical history before assessing the sitting diastolic blood pressure (DBP) and systolic blood pressure (SBP). The formula to compute the TyG index is ln [TG (mg/dl) × FBG (mg/dl)/2] (23).

### 2.4 Test for H. pylori

H. pylori was detected by ^13^C or ^14^C urease breath tests (24). The procedure for the ^13^C breath test involved collecting the initial breath sample under fasting conditions, followed by ingestion of a ^13^C urea capsule. After a 30 min period, another breath sample was obtained and both samples were analyzed using the instrumentation. In the ^14^C breath test, the steps included consuming a ^14^C urea capsule, adding water, waiting 15 min, gently blowing air through the conduit for 1–3 min, and analyzing the results by inserting the gas collection card into the detector.

### 2.5 Statistical analysis

Continuous variables that followed a normal distribution were assessed using the *t*-test, while variables deviating from normality were evaluated using the Mann–Whitney test. A chi-squared analysis was conducted for the categorical data. Multivariate logistic regression was used to examine the relationship between H. pylori and carotid plaque. Furthermore, restricted cubic spline (RCS) analysis was also used to investigate the linear or nonlinear relationship between the TyG index and carotid plaque, with nodes placed at the 10th, 50th, and 90th percentiles. Data analysis was conducted using R software (version 4.1.3), and statistical significance was determined at a two-sided *P*-value < 0.05.

## 3 Results

### 3.1 Baseline characteristics

This cohort study included a total of 5,994 healthy check-ups with a mean age of 50.7 years and a mean follow-up of 1.67 years. Of all participants, 1,948 (32.5%) were female and 4,046 (67.5%) were male, with a 42.6% rate of H. pylori infection at first physical examination. The clinical characteristics of all individuals were shown in Table 1.

**Table 1:** Baseline characteristics of all physical examination populations.

Variables	H. pylori-negative (*n* = 3,439)	H. pylori-positive (*n* = 2,555)	*P* value
Gender (*n*, %)			0.043
Female	1,154 (33.6)	794 (31.1)	
Male	2,285 (66.4)	1,761 (68.9)	
Age (years)	50.22 ± 0.18	51.30 ± 0.21	<0.001
Smoke (*n*, %)			0.009
No	2,422 (70.4)	1,719 (67.3)	
Yes	1,017 (29.6)	836 (32.7)	
Drink (*n*, %)			0.506
No	2,518 (73.2)	1,851 (72.4)	
Yes	821 (26.8)	704 (27.6)	
Triglycerides (mmol/L)	1.95 ± 1.65	2.00 ± 1.71	0.241
Total cholesterol (mmol/L)	5.06 ± 0.96	5.06 ± 0.95	0.990
High density lipoprotein (mmol/L)	1.40 ± 0.30	1.38 ± 0.28	0.065
Low density lipoprotein (mmol/L)	2.71 ± 0.72	2.69 ± 0.69	0.446
Diastolic blood pressure (mmHg)	77.26 ± 11.70	77.45 ± 11.52	0.548
Systolic blood pressure (mmHg)	127.98 ± 17.47	128.48 ± 17.71	0.282
Fasting blood glucose (mmol/L)	5.43 ± 1.47	5.49 ± 1.54	0.124
Glycated hemoglobin A1c (%)	5.88 ± 0.89	5.93 ± 0.94	0.024
Carotid plaque (*n*, %)			0.001
No	2,456 (71.4)	1,723 (67.4)	
Yes	983 (28.6)	832 (32.6)	

### 3.2 Univariate analysis of risk factors for carotid plaque

**Figure 1:**
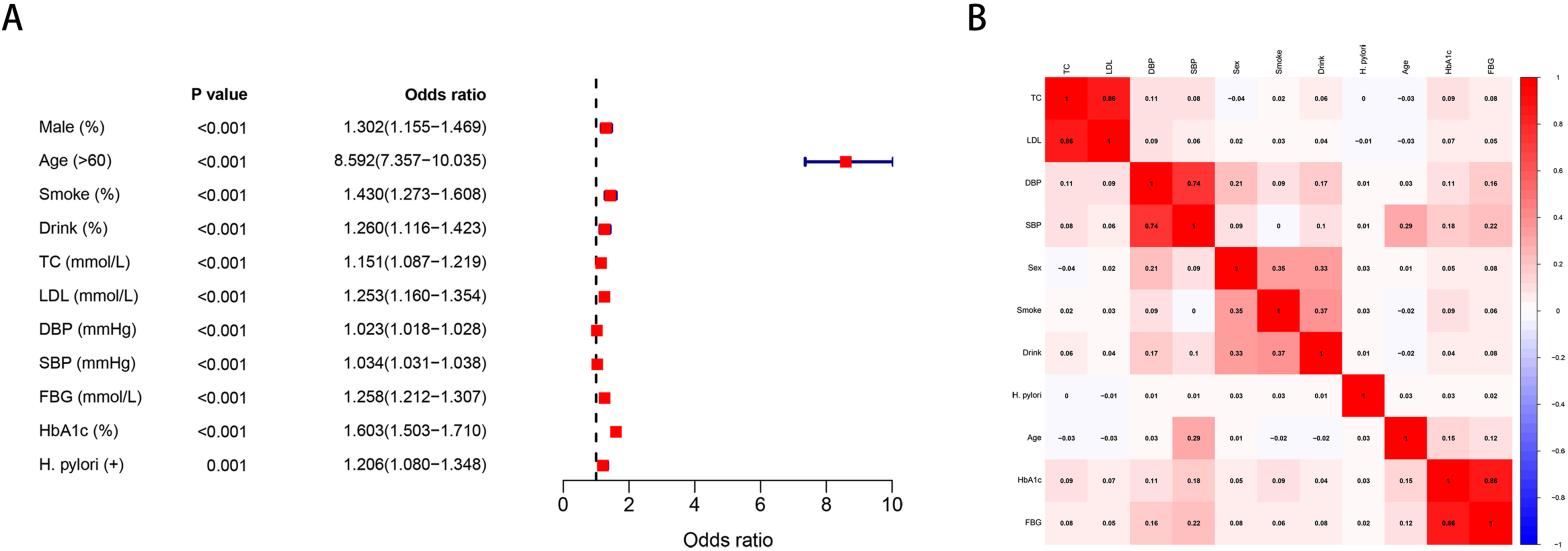
**(A)** Univariate analysis of risk factors for carotid plaque. **(B)** Correlation of various risk factors.

In univariate analysis, H. pylori was associated with an increased risk of carotid plaque formation (OR = 1.21, *P* = 0.001). In addition, male, age >60, smoking, drinking, TC, LDL, DBP, SBP, FBG, and HbA1c were also important risk factors for carotid plaque (Figure 1A). The correlation of each risk factor was shown in Figure 1B.

### 3.3 Multivariate logistic regression analysis of H. pylori and carotid plaque

To control for the influence of confounder, multiple regression analyses were performed for smoking, drinking, blood pressure, lipids, and glucose, in addition to sex and age, respectively. In all covariate adjusted multivariable regression models, H. pylori remained a notable risk factor for carotid plaque (Table 2).

**Table 2:** Multivariate logistic regression analysis of the relationship between H. pylori and carotid plaque.

	OR (95% CI)	*P* value
Model 1	1.16 (1.03–1.31)	0.018
Model 2	1.15 (1.02–1.30)	0.026
Model 3	1.17 (1.03–1.31)	0.014
Model 4	1.16 (1.03–1.31)	0.017
Model 5	1.14 (1.01–1.29)	0.037
Model 6	1.14 (1.01–1.30)	0.036

Model 1 was adjusted for age, sex.

Model 2 was adjusted for age, sex, smoke, drink.

Model 3 was adjusted for age, sex, TC, LDL.

Model 4 was adjusted for age, sex, DBP, SBP.

Model 5 was adjusted for age, sex, FBG, HbA1c.

Model 6 was adjusted for age, sex, smoke, drink, TC, LDL, DBP, SBP, FBG, HbA1c.

### 3.4 The longitudinal association between H. pylori and carotid plaque

**Figure 2:**
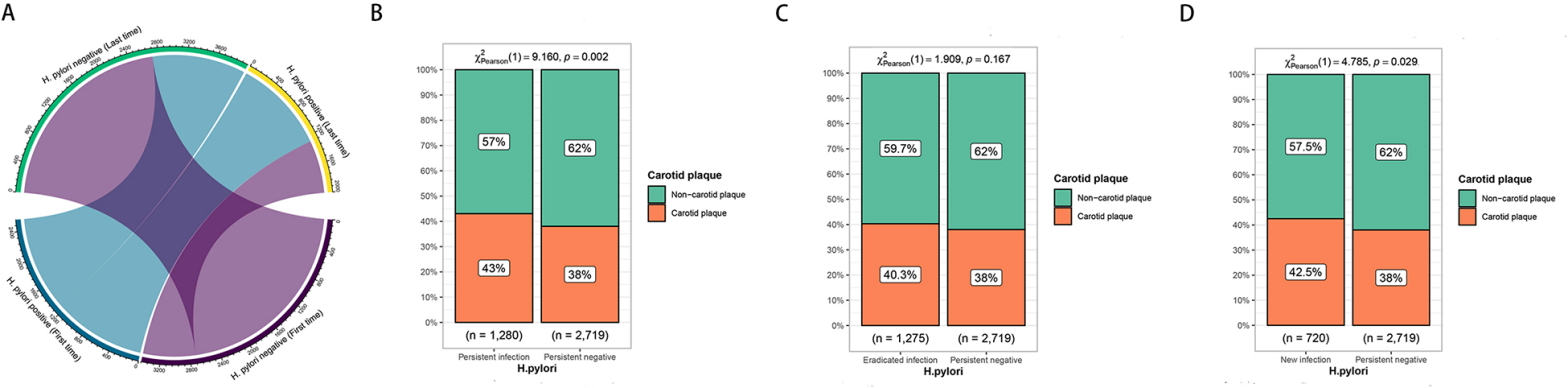
**(A)** Changes in the status of first and last H. pylori infections. **(B)** Difference in carotid artery prevalence between persistent infection and persistent negative. **(C)** Difference in carotid artery prevalence between eradication infection and persistent negative. **(D)** Difference in carotid artery prevalence between new infection and persistent negative.

The groups were categorized as persistent infection, persistent negative, new infection, and eradicated infection based on H. pylori status at the initial and final physical examinations. Changes in H. pylori infection status from the first to the last examination were observed during the follow-up, as depicted in Figure 2A. The risk of carotid plaque was significantly higher in the new infection and persistent infection groups compared to the persistent negative group (Figures 2B–D).

### 3.5 Differences between persistent negative and persistent infection groups

**Figure 3:**
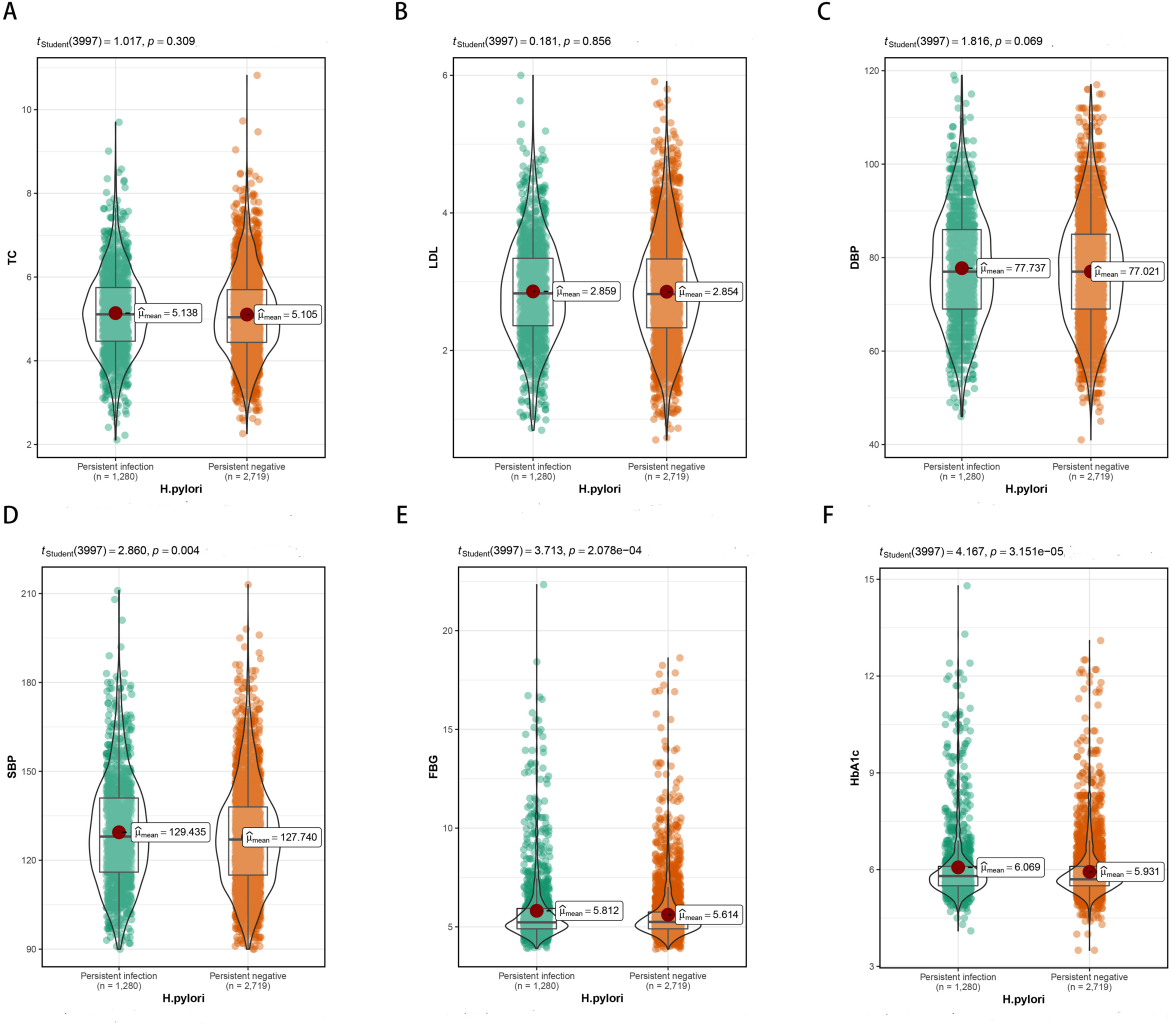
Differences in TC, LDL, DBP, SBP, FBG, HbA1c between persistent infection and persistent negative groups. **(A)** Differences in TC. **(B)** Differences in LDL. **(C)** Differences in DBP. **(D)** Differences in SBP. **(E)** Differences in FBG. **(F)** Differences in HbA1c.

We further analyzed the differences of other clinical variables between the persistent positive and negative groups. Within the persistent infection group, SBP, FBG, and HbA1c showed noteworthy increases compared to the persistent negative group, whereas no significant differences were detected in blood lipids (Figure 3).

### 3.6 The role of TyG index in carotid plaque

**Figure 4:**
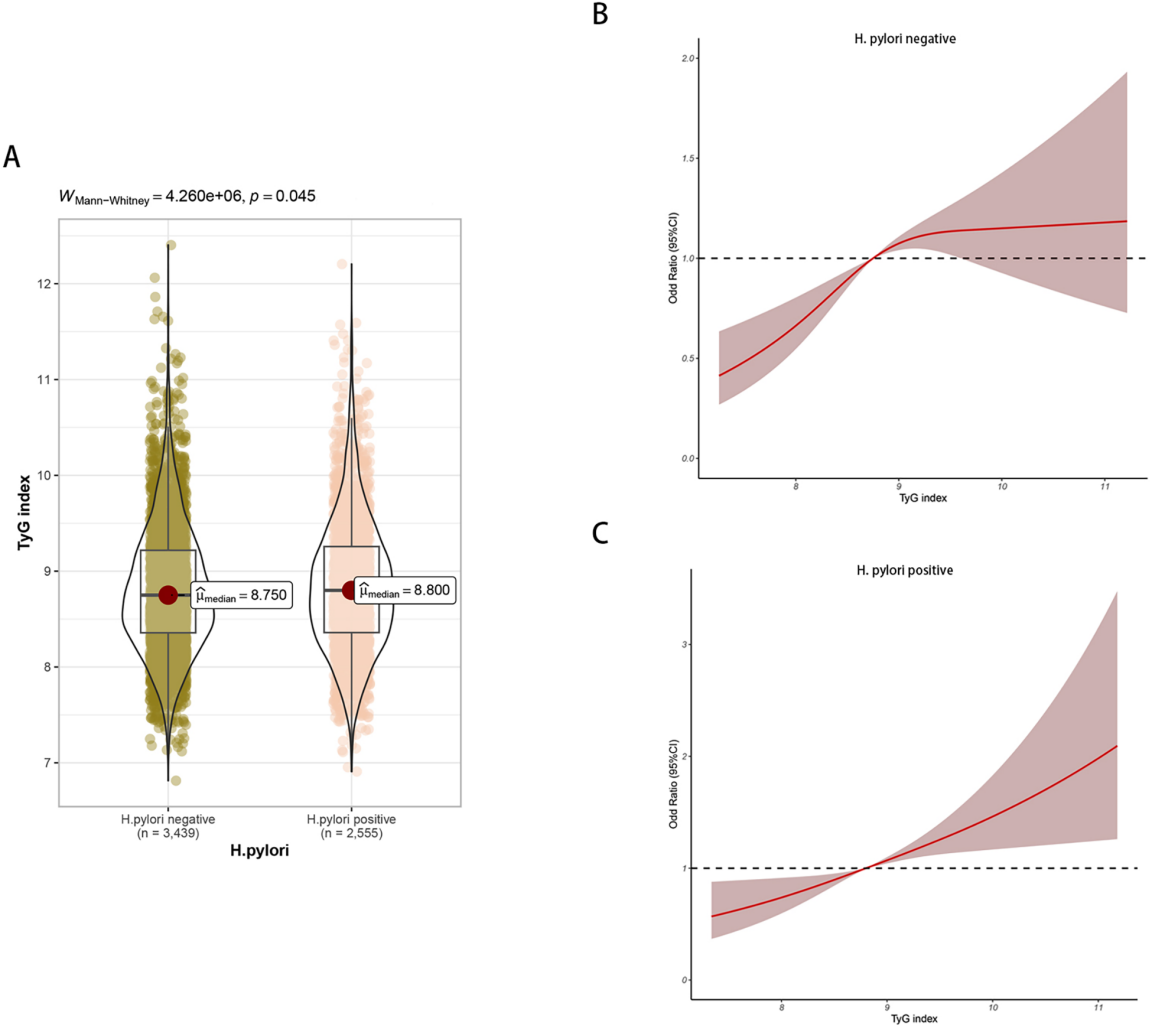
**(A)** Differences in TyG index between H. pylori positive and negative groups. **(B)** Non-linear relationship between TyG index and carotid plaque in H. pylori negative group. **(C)** Non-linear relationship between TyG index and carotid plaque in H. pylori positive group.

Differences in TyG index were found in H. pylori negative and positive groups (Figure 4A). In the H. pylori negative group, following adjustments for factors such as sex and age, the RCS model displayed a nonlinear relationship between the TyG index and carotid plaque (*P* = 0.013), as depicted in Figure 4B. In the H. pylori positive group, the RCS model showed no nonlinear relationship between the TyG index and carotid plaque (*P* = 0.732), Figure 4C; the risk of carotid plaque significantly increased with higher TyG index.

## 4 Discussion

Cardiovascular disease stands as the leading cause of mortality globally, with a rising incidence annually observed in both developed and developing nations (25, 26). Atherosclerosis serves as the fundamental pathological mechanism underlying cardiovascular disease, wherein carotid plaque represents a manifestation of this condition (27). It has been reported that infectious diseases may also be associated with the development of atherosclerosis (28, 29). To date, an increasing body of research has substantiated the significant correlation between H. pylori infection and cardiovascular events (30). In individuals diagnosed with acute coronary syndrome, H. pylori serum positivity is directly related to the short-term incidence of adverse cardiovascular events (31). Furthermore, research has shown that H. pylori infection can increase the risk of cardiovascular events by 3 to 4 times; nevertheless, eradicating H. pylori does not diminish the risk of cardiovascular events (32). Carotid plaque is a significant risk factor for cardiovascular events; however, the association between H. pylori and carotid plaques is still a topic of debate. In individuals under 50 years old, H. pylori infection may increase the risk of carotid atherosclerosis (33). Conversely, in a separate study of 14,588 healthy individuals, no link was established between H. pylori and heightened carotid intima thickness (34). Across multiple studies, H. pylori infection consistently correlated with increased CIMT (35, 36). Yet, most of the above studies, which were performed as cross-sectional studies, lacked evidence that long-term H. pylori infection affects carotid plaque. In our study, H. pylori infection, male, age >60, smoking, drinking, TC, LDL, DBP, SBP, FBG, and HbA1c were confirmed as significant risk factors for carotid plaque by univariate analysis. After controlling for confounding factors, our study revealed that H. pylori posed a risk factor for the development of carotid plaque formation through multivariate logistic regression analysis. Moreover, our cohort study provided additional confirmation that long-term H. pylori infection was associated with an increased risk of carotid plaque formation.

Long-term atherosclerosis leads to chronic accumulation of occlusive plaque in blood vessels, which eventually leads to narrowing of blood vessels (37). Previous studies have confirmed the involvement of LDL, TC and other lipids in the formation of atherosclerosis, which was consistent with our study (38, 39). H. pylori infection can affect lipid metabolism through various mechanisms (40, 41). Nonetheless, the mechanism by which H. pylori induces carotid plaque has not been clarified. In our study, we observed long-term H. pylori infection, which did not significantly impact the changes in LDL and TC levels. However, the persistent effect of H. pylori infection may result in alterations in blood pressure and blood glucose levels, potentially contributing to the development of hypertension and diabetes. Several research have reported that H. pylori can induce chronic inflammatory and immune response in the gastrointestinal tract and that some inflammatory cells such as leptin and tumor necrosis factor alpha (TNF-α) are involved in this inflammatory and immune response (42, 43). High levels of TNF-α and low levels of leptin can increase IR (17, 44). Abnormalities in the secretion of these associated hormones can further affect diabetes susceptibility (45). Similarly, various pro-inflammatory and inflammatory mediator release perturbations can induce endothelial dysfunction, which leads to arterial blockage, resulting in arterial hypertension and artery-related disease (46, 47). In addition, IR is considered to be one of the hazard factors for atherosclerosis (48). The TyG index, as a surrogate for IR, has been increasingly studied to confirm the association with carotid plaque (49, 50). In our study, differences were found in the effect of IR on carotid plaque between groups with and without H. pylori, suggesting a potential role of H. pylori in modulating the influence of IR on carotid plaque development. Therefore, we speculate that H. pylori is more likely to induce carotid plaque formation by influencing systemic inflammation and immune response rather than by affecting lipid changes.

Our cohort study confirmed the association between long-term H. pylori infection and an increased risk of carotid plaque. Nevertheless, the study exhibits several limitations. Firstly, it was conducted at a single center, suggesting a need for a multicenter longitudinal study to bolster the findings. Secondly, ultrasound is highly sensitive to carotid plaque, but there is no further grading of carotid plaque severity. Thirdly, despite employing various adjustment methods for confounding factors, the study may still be influenced by unaccounted potential variables. Fourthly, while the new infection group had a higher risk of carotid plaque compared to the persistent negative group, the exact duration of infection in the new infection group was unclear and might require more evidence to confirm this finding. Furthermore, the precise mechanism through which H. pylori impacts carotid plaque formation requires deeper investigation.

## 5 Conclusion

H. pylori is a risk factor for carotid plaque, with a long-term infection associated with an increased risk of carotid plaque formation. Moreover, the pathway by which H. pylori infection contributes to carotid plaque formation might be linked to blood pressure, blood sugar levels, and IR. Eliminating H. pylori could carry significant benefits for cardiovascular disease prevention.

## Data Availability

The raw data supporting the conclusions of this article will be made available by the authors, without undue reservation.
